# Valedictory Address to the Graduating Class, Rush Med. College, Feb. 17, 1874

**Published:** 1874-04

**Authors:** De Laskie Miller

**Affiliations:** Prof. of Obstetrics


A MONTHLY RECORD OF

*Medicine, Surgery and the Collateral Sciences.*

EDITED BY J. ADAMS ALLEN, M.D., LL.D.; AND WALTER HAY, M.D.

---

VOL. XXXI.—APRIL, 1874.—NO. 4.

---

### Original Communications.

ARTICLE I.—*Valedictory Address to the Graduating Class, Rush Medical College, Feb.* 17, 1874. By DE LASKIE MILLER, M.D., Prof. of Obstetrics.

Standing in the centre of an extended plain, the tallest man can discover objects only at a limited distance. Should he be surrounded by undulations of the surface, the radius of his vision would thereby be shortened, and its area circumscribed. Would he look out and over the landscape, one thus situated must seek some elevation; he must ascend to the lofty summit, though to reach it requires toilsome exertion. Then only does the beauty of the varied scenery presented to his vision around and below him, repay his effort.

As in the physical world, so in many respects is it in the intellectual. The uneducated man sees no harmony or relation in the varied phenomena of nature. And if surrounded as he usually is by superstition, the unveiling of the forces of nature excites in his mind rather the feelings of apprehension and dread. He cannot look beyond the cloud, nor see by the lightning's glare, nor above the thunder hear a "still small voice." It is not till one thus situated has ascended from the low plane of ignorance, that fear will give place to any real pleasure, in the contemplation of nature or of nature's works.

The progress of the scholar has been allegorically compared to one making a difficult ascent of the steep mountain-side—"The hill of Science"—up which the toilers make slow and unequal progress. Some outstrip their companions for a time, but relax their efforts and fall behind when only part way up. A few, though they advance slowly, finally, by perseverance, reach the summit, and then realize a consummation of the pleasures of hope, as revealed in a knowledge of the sciences which they now may read in the volume of nature open before them.

As he who from a favorable height views the varied landscape, with its hills and valleys, river and lake, city and hamlet, presenting variety and beauty, but also a complexity amounting almost to confusion: so with the scholar, when retrospecting the subjects which have, individually and in detached portions, for so long a time occupied his attention. So diverse and even antagonistic do they seem in their nature, that to reduce them to a unity, and bring their manifestations into harmony, may well appear impossible. For does he not observe in the phenomena of nature the almost endless variety and complexity of her forces? How diverse in mode and result!

By partial views he regards light, heat, gravitation, electricity, magnetism, sound, as distinct entities. The operation of some *one* or more of these forces he sees manifested in the dew-drop sparkling in the rays of the morning sun, or in the brilliant hues of the flower on the mountain-side, or in the ponderous force that has lifted the summit of that mountain above the clouds—in the lightning's flash and attendant thunder—in the movements of planets through ether. Could anything seem more demonstrative of *diversity of forces*, subject to *different laws?*

The sum of human knowledge, when thus scanned, appears broken and fragmentary. The actual procedure of nature differs diametrically from man's method necessary to arrive at a knowledge of it. From primordial unity *her* processes radiate in multiplied combinations. Man must seize upon each separate development, group by comparison and distribution, and slowly and painfully explore his way to the parent stem.

Since the discovery of the law of gravity, more than two hundred years ago, no scientific achievement has been so fruitful in results as the determination of the mechanical equivalent of heat. A body of a given weight, falling through a given distance, suddenly arrested, produces a definite elevation of temperature. The knowledge of this simple fact has wrought a marvelous change in all our conceptions of matter and force, and gives promise of results which the imagination fails to grasp. It is now considered that heat, motion, light, electricity, magnetism, chemical attraction, and mechanical work, are all but exhibitions of one and the same power, acting through matter. The molecules of matter variously stirred by this all-pervading force are thrown into waves, which impinge against our senses, and the motion thus communicated to our nerves, impresses us as heat, sound, light, etc., according to the rapidity and breadth of the undulations. If an undulation of one sort is interfered with, another immediately succeeds, of exactly the same strength. Light transforms itself into chemical action, this into heat, and heat into motion. All these modes of motion are not only mutually convertible, but they may also be turned into mechanical work. Force is a constant energy, and has been since the world began, never increasing nor diminishing in absolute value. Like a stream, now flowing in an uninterrupted channel silently and imperceptibly, now thrown into gentle undulations as it comes into contact with the subtle forms of matter, and now giving striking display of its power, as it meets with greater resistance. Light, sound, heat, the invisible flow of the terrestrial magnetic currents, as well as the aurora and zodiacal light, the circulation in plants and animals, are but modifications of a single force!

A professor of natural science once received from a member of his class the following question, "What would be the result, if an *irresistible* force should come in contact with an *immovable* body?" The professor, not suspecting that he was being quizzed, went into an elaborate argument to explain what would happen, with this conclusion, "The *irresistible* force would be partially obstructed, while the *immovable* body would yield a little, and finally they would settle at rest!" Query, was the professor posted in the modern doctrine of the persistence of force?

From the unity of force, a single step leads us to the comprehension of the unity of matter. The whole tendency of the revelations of modern science is to do away with the notion of an indefinite number of primary elements, and therefor to substitute a smaller and still smaller number of primitive forms of matter. By changes in the mode of aggregation of the atoms, which changes depend on the degree and kind of motion with which they are surrounded, matter appears to us under certain definite forms—as water, earth, iron, etc. From the results of analyses already made, we are justified in the inference, that bodies now believed to be elements must yet yield to the quest of the scientist the fact of their inherent compound composition. What revelations may we not hope for from our modern improved means of interrogating nature? Results may be looked for, more startling than the dreams of the alchemist; results which warrant the conclusion that *variety* of form and *unity* of substance, the evolution of the complex from the simple, is the fundamental principle of nature. Carried further, this view renders probable the position, that, pervading the vast domain of nature, occupying interstellar space, an *ether* exists in a state of *motion*. The ethereal atoms form societies, which are the molecules of bodies—a body is a collection of these societies of molecules. Between these atoms—molecules and bodies—exchanges of motion take place, which give rise to the phenomena of light, heat, gravity, etc. Pursuing the thought to this point, the scientist is led to exclaim, "Grant us, then, the *atom* and *motion*, we show you the *universe!*"

This doctrine is presented as the lesson of to-day. By experiment and logic we are urged to adopt the conclusions. When our assent to this has been obtained, we are bid to step boldly across the line that divides inorganic from the organic, and note the wonderful in this new domain! What force raises the forest against gravitation, till the branches reach the clouds! Estimate the weight of the giant oak, and all doubt of the potent energy of nature will be dissipated. In the vegetable kingdom, the operation of heat, as the dynamical agency to which the phenomena of growth and development are to be referred, is well seen in the process of germination. But this process is by no means a simple one. The nutriment stored up in the seed is, in great part, in the condition of *insoluble starch*, and this must be brought into a *solu-*

*ble form* before it can be appropriated by the embryo. By the *diastase*, starch is converted into *dextrine*, and then into sugar; the dextrine and sugar combine with the albuminous and oily compounds, and form the protoplasm, from which the tissues of the young plant are derived.

This account of the uses of starch in vegetation differs, in some particulars, from that given by the student under examination for his degree, who, when asked, "What are the uses of starch in germination?" answered, "The uses of starch in the German nation and elsewhere are for dressing linen, and for other laundry purposes."

Now, it can be easily shown, experimentally, that the rate of growth in the germinating embryo is so closely related (within certain limits) to the amount of heat supplied, as to place its dependence upon that agency beyond reasonable question, so that we are entitled to say that *heat*, acting through the germ, supplies the constructive force by which the vegetable fabric is built up. The correlation between heat and the organizing force of plants, is not less intimate than that which exists between heat and motion. The special attribute of the vegetable germ is the power of utilizing, after its own particular fashion, the heat which it receives, and of applying it as a constructive power to the building up of its fabric after its characteristic type.

Advance to a higher plane, and these identical forces are still met, and may be appreciated in the complex functions peculiar to animal organization.

The primitive cell, like that of the vegetable, is formed out of the inorganic world, and in both a series of motions succeed each other, according to a fixed order, obeying the law of molecular mechanics; *vital* energy, then, is the resultant effect of all other forms of force. The organism is subject to, and readily responds to all physical influences. Heat, light, and motion, are indispensable to life, and to the inert germ they, under proper conditions, impart the required energy to perfect and mature the organism. No living body can originate power: it can only convert the stored force derived from food. The force of the animal organism is derived from the vegetable, and that, in turn, from the mineral, through chemical interchanges.

As vibratory motion, in the animal organism, finds a resultant in integral motion, so mechanical motion may be resolved, in the organism, into vibratory motion. Modes of external and internal friction are excitations which stimulate and quicken vitality. There is no part of the vital economy of any organ of growth, increase or decay—no change in any organic expression, but is the result of a movement in which the entire vitality has participated, directly or remotely, and in all the parts harmoniously.

The law of the unity and the persistence of force transcends experience by underlying it. Its dominion is not limited to physical phenomena: it prevails equally in the world of mind, controling the faculties and processes of thought and feeling. The star-suns of the remote galaxies dart their radiations across the universe; and although the distances are so profound that hundreds of centuries may have been required to traverse them, the impulses of force enter the eye, and, impressing an atomic change upon the nerve, give origin to the sense of sight. Star and nerve tissue are parts of the same system. Stellar and nerve force are correlated; more, sensation awakens thought and kindles emotion. So that this wondrous dynamic chain binds into living unity the realms of matter and mind through measureless amplitudes of space and time.

Emotion and ideas display outward phenomena, which are subject to the laws of thermo-dynamics. The operations of the mental and emotional faculties are accompanied by a change of temperature in the brain. May not, then, the greatness of an idea and the strength of an emotion be measured, and their mechanical equivalent established?

Upon premises like these, *one law in nature* is introduced as the radical fact in the creed of to-day, which is interpreted to mean that this all-pervading and unchanging *law* is the "*fons et origo*" of all the wonders that salute us on every hand, admitting no intelligence or will superior to it.

(Lest my friend here (Rev. Dr. Sullivan) may begin to feel anxious about the orthodoxy of a member of his flock, I stop to say that these Unitarian views are advanced in a scientific sense, not in a theological.)

I regard the facts established by science as comparable to a few pebbles, gathered by finite minds on the shore of the infinite ocean of truth. What, then, are the mysteries still hidden in the bosom of the mighty unexplored? How far transcending all stretch of thought, that unknown and Infinite cause of all things?

The facts which we think we comprehend, we are attempting, as children string their beads, to arrange and classify, that we may study their relations with each other. It is fortunate for us that this string which we call *law*, is not an iron chain, binding the Infinite to blind chance, but a golden thread held by the hand of the Creator.

I see behind these phenomena, and upon which they depend, a *force*, that knows no difference between great and small, between ponderous and buoyant—a *force* that moves all things in harmony with its own will; that requires no greater effort to create a planet than to round a tear—a force which can reside only in Omnipotence, which we name God!

To Sir William Hamilton is attributed the following sentiment, which is reflected by gilded letters on the wall of one of the lecture rooms of the University of Edinburgh: "On this earth there is nothing great but man; in man there is nothing great but mind." Do I violate any rule of ratiocination when I assume the origination of all power in mind? Recall a sight frequently seen from our city: two noble ships, well freighted, sailing on right lines, but in opposite directions; if left to the force of the winds, would they not drift to certain destruction? Could the magnificent system of railways in our country ever have been constructed, independent of the *mind* of the engineer? What power, in two short years, could raise from ashes a city, which, in style and elegance, eclipses all others, but the *intelligence* of our citizens? And further, there are grounds for the belief that the phenomena of the material universe are the expressions of a mind and will of which man's is the finite prototype. To admit this, will be to admit the existence of the supernatural, always working in and through the natural. Force ceases to be a blind attribute of matter, and becomes a living, active principle, spiritual in its character, to which the human spirit turns evermore in solemn and mysterious worship.

The argument has been extended even beyond the point which we have reached. Carried to its logical sequence, it is affirmed that the theory of *evolution* is established. The conclusions succinctly stated, stand thus: The origin of species is the result of the ever varying manifestations of force. Vital force is not an independent energy; the modes of interchange are those of motion and primary force. Organic growth is affected by moisture, light and heat-motion. All organic as well as inorganic interchange is atomic. The axes of crystals even represent unequal resultants of force: so resultants of organic interchange are represented by unequal axial directions. Interchange is solicited in one direction more than another, and the resulting figure of growth is elongated in that axial direction. So that organic interchange is greatest in the direction of most motion, and follows the paths of least resistance. From a dynamic point of view, natural selection is the evolution of life, along lines of least resistance. The preservation of varieties that succeed better than their allies in coping with surrounding conditions, is the continuation of vital movement in those directions where the obstacles to it are most eluded. On this is based the doctrine of "the struggle for existence, and the survival of the fittest."

This doctrine excludes creation and theism. The great work of nature by evolution culminates in the production of man. In reaching his present high estate of powers and endowments, man represents the sum of all the creative energies from primeval time; and is an improvement only, upon all that has preceded him. Man civilized is more perfect than the ape, more perfect than the savage.

Is this the *true* account of man's genesis? If it is, it must and will be found in accord with religion and revelation. What we call the *laws of nature*, are but the modes of expression of the Divine Will. Time is an unessential element in the fruition of God's great plan, for with Him "a day is as a thousand years, and a thousand years as a single day." I am anxious only as to the *truth* of the theory of evolution. Manifestly much remains to be learned, by finite minds. As presented by its advocates, the doctrine of evolution is *incomplete* or *untrue.*

I perceive in the long road of evolution numerous chasms, which remain to be filled up, or bridged over. In this chain of succession numerous links are wanting to complete its continuity and strength before it can bind as a law. A few are as follows, *e. g.:* First, material and force with which evolution shall begin. Whence come they? Are all things made out of nothing and by nothing? or are we *idealists*, and choose to believe in the non-existence of matter, and thus play with language? Though we say that man descended from the ape, the ape from an amphibious animal, this from a fish, and this, in turn, through long successions from the protean Rhizopod, which performs the functions of vitality without organs; laying hold of its food without members, swallowing it without a mouth, digesting it without a stomach, assimilating it without absorbent vessels, or a circulating system, moving without muscles—a fragment separated giving origin to a new and independent being, —still we are as far from a satisfactory answer as when we started! Second, Where shall the link be inserted that connects animals and vegetables? Third, The gap that yawns between the species is as broad to-day, as it was before Darwin undertook to fill it. Fourth, It has not been proved that any animal, by any force in itself, can rise to the self-conscious and reasoning nature of man. The intelligence of animals is a closed circle, ever returning into itself: while that of man is progressive, inventive and accumulative. Fifth, Nor can the gap between the religious and moral sentiments of man and the instincts of brutes be filled by the miserable ape.

The alternative is the doctrine, that "God formed man of the dust of the ground, and breathed into his nostrils the breath of life, and man became a living soul." This doctrine carries with it the inference of separate creations. I suppose it to mean that all things have been produced by the Supreme creative will, either directly or mediately, through the agency of the forces and materials of his own providing. This does not imply that creation was miraculous, in the sense of being contrary to law. It does not contradict the idea of succession. There is no necessity that the process should be instantaneous and without progression. It does not imply that all kinds of creation are alike. Man's creation was in perfect harmony with the laws of procedure, which the Creator had established for his own operations. May not creation and derivation be complementary of each other? Is there any more difficulty in believing in the creation of ten millions of species (vegetable and animal), than in one out of nothing.

In all this matter we represent the man standing in the centre of an extended plain. The most cultivated and exalted intellect can discover the relation of facts only to a limited extent. *We* see no relation between phenomena, but just beyond our intellectual horizon all may be manifested. In our view, the chain may appear broken; there, the links may be supplied. To us, chasms appear impassable; there, no irregularities exist, for—

> " All are but parts of one stupendous whole,
> Whose body nature is, and God the soul."

In conversation upon the merits of medical practice, one of the most learned and eloquent divines of this country said to me, " In my mind the question is settled. My father's family has been raised under the science and skill of regular physicians; my own has been thus far, and I entertain no thought of change. I am willing to accept results." In like manner I hold to the teachings of the Bible upon this point. From this source we obtain a more satisfactory solution of the subject than has been presented elsewhere. From revelation only, do we obtain any just conception of the attributes of an Artificer capable of producing such a work as man. "So noble in reason; so infinite in faculties; in action so like an angel; in apprehension so like a God; the beauty of the world, the paragon of animals—surely he must be allied to the Deity."

We are in the habit of measuring the greatness and the wisdom of the universe by the duration and profit it promises to our own race. A Roman sword or vessel from Nineveh awaken in us the conception of grey antiquity. We gaze upon the remains of Egypt and Assyria with silent astonishment. With an effort, we carry our thoughts back so far. Still, the human race must have existed for ages, and multiplied itself, before the pyramids were raised! We estimate the duration of human history at 6,000 years; but what is this in comparison with the time during which the earth carried successive series of rank plants, mighty animals, and no men. The history of man is but a short ripple on the ocean of time.

During the lapse of these cycles, there has been no time when to live was so great a boon to intelligent creatures as the present. Though it be a time of restless activity; of conflict of minds; of aspirations after the higher and better—it is a time, also, of progress, of invention, of improvement. Let the struggle proceed; it will surely issue in the survival of the fittest.

Coming out of this universal unrest, already we have innumerable appliances to aid in human efficiency, the conversion of force in a thousand ways doing the work of human hands—steam for our motive power, lightning for our messenger, by which distant nations are made neighbors, effecting by concentration an increased efficiency of thought. A new idea or invention, started on any part of the globe, instantly becomes the property of all, and is at once assimilated into the accumulated mass of intellectual capital. Thus, by the telegraph wire—the *chorda vitalis* of mankind—the minds of all peoples act in harmony, responsive as the brain of an individual, in moving forward the car of progress.

Though invention and execution have added immensely to our resources for social, domestic and economic comfort and utility, the demands upon science are not yet canceled ; nor will they be, till we have gained better and cheaper motive powers than steam ; till we may travel from continent to continent on submarine railways, or by flying or ballooning through the air ; till man can call down rain at will, when crops may be increased five-fold ; till artificial light for domestic use shall be as cheap and abundant as water ; till human life is protected from the dangers of explosions ; till death's high carnival on land and sea is ended, by utilizing force, so that collisions become impossible, by rendering trains and ships repellant, preventing contact.

To members of the medical profession, the world is indebted for many of the useful inventions of life. Still greater achievements await well-directed efforts. I have an abiding faith in the unlimited possibilities of medical science. The day will come when physicians shall be familiar with the chemistry of diseases ; when they shall know the exact poisons that produce them, and their antidotes ; when they shall look upon the cure of maladies as simply a series of chemical problems and formulas ; when they shall melt down all calculi and necrosed bone chemically, and not remove them by surgical operations ; when hemorrhage shall be arrested, not mechanically, but by the simple application of gases and washes ; when wounds shall be swiftly healed by first intention ; the ravages of tubercle shall be stayed ; fevers and inflammations shall be blotted out ; when all morbid growths may be melted down ; cancer cured ; when all morbific organic germs may be destroyed ; when contagion may be annulled—and thus the average duration of human life shall be lengthened, so that the ancient prophecy may begin to be fulfilled : " The child shall die an hundred years old." With *force*, *matter*, *mind*—the trinity of humanity—what may not be accomplished?

Standing on this high plane of improvement and possibilities, imbued with the spirit of the age, we may serenely contemplate the history, achievements and prospective triumphs of the medical profession. In its broad catholicity all are made recipients of its kindly offices. Especially when the dark cloud of disease is settling down to envelop, there are none so high, though the brow be pressed by a glittering crown, that they do not petition it for relief; there are none so lowly, though possessing only poverty and its deprivations, but bless it for its benefactions.

From this standpoint how puerile and petty seem the discussions that have sometimes marred the life and character of the disciples of Esculapius!

How simple, to apply no stronger epithet, are those who restrict themselves to portions only of the resources of our art, and boast of belonging to a distinct school! A distinct school, forsooth! There is but *one* school of medicine, as there is but *one* science of medicine, which embraces all sciences. Cavilers at scientific medicine are not peculiar to our day. They have, in one form or another, from the earliest days, arrogated to themselves the honorable title of physician, and often disparaged science. Such should be warned by the fate of their great prototype, of whom Milton wrote:

"He fell
From morn to noon, from noon to dewy eve,
And was headlong sent
With his industrious crew to build in ****" = the pit whose perfume is that of their specific for psora.

History is burdened with the detail of the successive and ignoble exequies of the sects who scoff at legitimate medical practice. And it shall ever be thus. Partial views are incomplete, therefore defective. Those who act on defective views may well be compared to bayous, which come to an abrupt termination, or to rivulets (and some are very small) that dry up in a brief time; while scientific medicine, like a noble river, ever increases in volume and power as it flows onward toward the infinite!

In looking back over this whole subject, is it not possible to see the beautiful harmonies of nature; that on her many stringed instrument *force* answers to *force*, like the notes of a great symphony; disappearing now in potential energy, and anon reappearing as actual energy, in a multitude of forms? Does not this wonderful unity and mutual interaction of force in the forms of inorganic nature, appear identical in the living forms of vegetable and animal life? That even that mysterious and in many aspects awful power of thought, by which man influences the present and future ages, is a part of this great ocean of energy. But here the great question rolls upon us, Is it only this? Is there not behind this material substance, a higher than molecular power, in the thoughts which are immortalized in the poetry of a Milton or a Shakespeare, the art creations of a Michael Angelo or a Titian, the harmonies of a Mozart or a Beethoven? Is there really no immortal portion separable from this brain-tissue, though yet mysteriously united to it? In a word, does this curiously fashioned body inclose a soul—God given, and to God returning? Here science veils her face, and bows in reverence before the Almighty. No word but His who formed us can break the awful silence. But faith comes in joyfully to accept that higher truth, which can never be the object of physical demonstration.

Graduates of 1874—Gentlemen:—I have made these remarks in your hearing as suggestive of thought. It may be a useful exercise of the mind for you to add to the argument and illustration. On an occasion so interesting as the present is to you, the topics touched upon are not inappropriate. No subjects are too broad or deep to claim your attention. Something more is expected from you than that you know how to estimate an ordinary dose of some sudorific or anodyne. You are expected to raise yourselves out of the groove, and go forth and study nature in her inviting fields.

By the act of the Faculty and Trustees of Rush Medical College, you are this evening raised to a higher plane than that on which you have traveled hitherto! You are placed on a level with the most gifted. You now start abreast with the most favored in this land. On you now devolve new duties and responsibilities. Said Lord Bacon, "I hold every man a debtor to his profession, from the which, as men of course do seek to receive countenance and profit, so ought they of duty to endeavor themselves, by way of amends, to be a help and ornament thereunto."

All that is worth striving for is before you. Your success will depend mainly upon your own exertions. Though you possess brilliant talents—genius, even—rely not on these; industry and perseverance are far more certain guaranties of success. To my knowledge, you have been faithful students. Let not the fatal notion take possession of your minds, that now study is ended; your application, on the contrary, should be redoubled. Keep in mind ever the allegory of the hill of science, press onward and upward, give way to no relaxation till your feet stand on elevations high as man has ever trod. Otherwise, your progress will be like the Arctic explorers on the ice-belt, who, while they walked ten miles toward the pole, drifted twelve toward the equator.

Demosthenes being asked the first requisite of the orator, replied, "action;" the second, "action;" the third, "action." Were I to define the requisites of the successful physician, it would be, read, read, read. Buy books and read them, then buy more books. True, some will prove well nigh worthless, but you will soon learn to distinguish fine gold from dross.

Gentlemen, for these many weeks we have floated delightfully together on the placid stream of time. To-night we drift apart, each to fill the space society may yield to him. You go hence bearing with you the kindest wishes and benedictions of the Faculty for whom I speak. We will ever rejoice in your success; we will ever sympathize with you in your trials.

Plato says, "Truth is the beginning of every good to the gods, and of every good to man; and he who would be blessed and happy, should be, from the first, a partaker of the truth, that he may live a true man as long as possible, for then he can be trusted."

From you we expect but one report. That in every relation of life—whether as men, as physicians, or as citizens—you have been *true* to yourselves, to the profession, to all.

I must not detain you longer. I would avoid the word

> "Farewell, a word that must be—and hath been
> A sound that makes us linger—yet, Farewell."

